# Sex-Dependent Effects of PM_2.5_ Maternal Exposure and Quercetin Intervention on Offspring’s Short Chain Fatty Acids

**DOI:** 10.3390/ijerph16224371

**Published:** 2019-11-08

**Authors:** Wei Liu, Yalin Zhou, Yong Qin, Yong Li, Lanlan Yu, Ruijun Li, Yuhan Chen, Yajun Xu

**Affiliations:** 1 Department of Nutrition and Food Hygiene, School of Public Health, Peking University, Beijing 100083, China; liuwei19560912@163.com (W.L.); zhouyalin2017@163.com (Y.Z.); qinyong0520@163.com (Y.Q.); 15895977095@163.com (Y.L.); putaoeternal@163.com (L.Y.); aiesecarrow@163.com (R.L.); cyhan_ss@163.com (Y.C.); 2 Beijing Key Laboratory of Toxicological Research and Risk Assessment for Food Safety, Peking University, Beijing 100083, China

**Keywords:** PM_2.5_, offspring, quercetin, short chain fatty acids

## Abstract

Short chain fatty acids (SCFAs) are produced by the colonic microbiota through fermentation. Influences of maternal PM_2.5_ exposure on SCFAs of the offspring have not been well understood. Additionally, studies of dietary intervention have not been carried out yet. Here we performed a study that dams were received PM_2.5_ and quercetin intervention during gestation and lactation. SCFAs in colon of dams and their offspring (on postnatal day 21 and 35) were analyzed using gas chromatography. For male offspring, when compared with the control group levels of acetic acid, butyric acid, and valeric acid were lower in the PM_2.5_ group (*p* < 0.05), however, levels of isobutyric acid and isovaleric acid were higher in the PM_2.5_ group (*p* < 0.05). For female offspring, as compared with the control group, propanoic acid was lower in the PM_2.5_ group, however isovaleric acid was higher in the PM_2.5_ group (*p* < 0.05). 100 mg/kg and 200 mg/kg quercetin intervention could inhibit SCFAs production of male offspring, especially in isobutyric acid and isovaleric acid (*p* < 0.05). 100 mg/kg quercetin intervention could upgrade the level of propanoic acid of female offspring (*p* < 0.05). Taken together, these results suggest that PM_2.5_ tracheal exposure during gestation and lactation could influence SCFAs of offspring. Quercetin administration might have the potential to offset the effects of mater PM_2.5_ exposure on SCFAs in the offspring to some extent. The above effects were showed in a sex-dependent manner.

## 1. Introduction

In recent years, ambient air pollutants, especially fine particulate matter 2.5 (PM_2.5_, aerodynamic diameter ≤ 2.5 μm), have received much attention. It has been reported that long- and short-term exposure to PM_2.5_ is harmful to human health. PM_2.5_ is related to adverse health outcomes, such as impaired pulmonary function, increased risk of cardiovascular, and cognitive deficit [1,2,3]. Epidemiological studies have reported that exposure to PM_2.5_ contributes to gastrointestinal diseases [4,5]. A study by Mutlu et al. demonstrated that PM_2.5_ exposure could significantly increase gut microbial diversity [6]. Changes in intestinal flora caused by PM_2.5_ exposure may affect the release of short chain fatty acids (SCFAs). 

SCFAs are produced by the colonic microbiota through fermentation [7]. The main substrates for fermentation are undigested carbohydrates [8]. SCFAs mainly refers to fatty acids with less than 6 carbon atoms in the carbon chain, including: acetic acid (C_2_H_4_O_2_), propionic acid (C_3_H_6_O_2_), butyric acid (C_4_H_8_O_2_), isobutyric acid (C_4_H_8_O_2_), valeric acid (C_5_H_10_O_2_), and isovaleric acid (C_5_H_10_O_2_). Among them, acetic acid, propionic acid, and butyric acid accounted for the highest proportion.

SCFAs play important roles in supplying nutrients and energy to the host. SCFAs have been shown to change cell proliferation and function, have anti-inflammatory and antimicrobial effects, alter gut integrity, and lead to amelioration of colitis [9,10]. Therefore, when SCFAs are affected, some organs and functions of the body may be affected. Acetate is utilized for lipogenesis in the liver and as a fuel source in the heart and brain once it enters the peripheral circulation [11]. Propionate is largely taken up by the liver for gluconeogenesis. Butyrate is the major fuel source for colonocytes [12], and therefore is beneficial for maintaining intestinal mucosal integrity and inhibiting the intestinal diseases such as inflammation and colorectal cancer [13].

Quercetin, a herbal polyphenol, may be derived from a variety of plants, such as apple, onion, tea, strawberries, and broccoli [14]. It has been identified to possess various biological activities, such as anti-oxidation [15], antimicrobial activity [16], wound-healing [17], anti-cancer activity [18] and immune-modulatory activity [19]. Shanthi et al. reported that polyphenols can influence the viability [20] and increased SCFAs (acetic acid, propionic acid, and butyric acid) production [21]. We suspected that quercetin supplement may have beneficial effects on SCFAs and alleviate the negative effects of PM_2.5_. 

As far as we know now, few studies have investigated the influences on SCFAs of offspring when mother was exposed to PM_2.5_ during gestation and lactation, and there has no nutritional intervention methods. To clarify the above issues we established mouse models involving pregnancy and lactation exposure to 15 mg/kg PM_2.5_ accompanied with the intake of quercetin. The biological effects on SCFAs in male and female offspring mice were explored. 

## 2. Materials and Methods 

### 2.1. Preparation of PM_2.5_ and Chemicals

The samples of PM_2.5_ were collected by a particulate sampler (TH-150C, Wuhan Tianhong Instruments Co. Ltd., Wuhan, China) in residential area of Beijing, China. The filter was agitated in ultrapure water. The solution was filtered through eight layers of gauze and centrifuged at 12,000 rpm for 20 min. The sediment was collected by a vacuum freeze drier (FDU-1100, Tokyo Rikakikai Co. Ltd., Tokyo, Japan). The dry PM_2.5_ powder was diluted in sterile phosphate-buffered saline (PBS) (0.01 M, pH 7.4) at a concentration of 15 mg/mL and kept at −20 °C before experiments. An extra control sample from unexposed filters was processed identically. Quercetin (Sigma-Aldrich, Shanghai, China, purity ≥95.0%) was respectively dissolved in 0.15% carboxymethyl cellulose sodium (CMCS) at a concentration of 10, 20 and 40 mg/mL. 

### 2.2. Dose Information

Our another article described the calculation method for PM_2.5_ dose [22]. Briefly, for a normal adult, respiratory minute volume is 6 L/min, and the daily respiratory volume is about 8.64 m^3^. In the same district, the daily highest average concentration of PM_2.5_ reached 0.43 mg/m^3^ [23], so the exposure concentration 3 days should be 0.185 mg/kg b.w. Considering extrapolation coefficient, the average concentration of PM_2.5_ exposure for each mice every 3 days was estimated to 18.5 mg/kg b.w. We found that maternal exposure to PM_2.5_ (15 mg/kg every 3 days) changed postnatal open-field behaviors in both gender, impaired spatial learning and memory in the female offspring [22]. In our previous animal experiments [14], we have found that PM_2.5_ could activate inflammatory reaction and oxidative stress level of pregnant mice at the concentration of 15 mg/kg. Herein, we take 15 mg/kg as the dose group. 

Although there is currently no reference intake of quercetin, quercetin supplementation has been shown to be beneficial in humans. According to the reported studies, the effective dose of quercetin was reported 8.3~60 mg/kg in humans [24,25]. The dosages in humans was equivalent to 51.5~372 mg/kg in rats. Therefore, we take 50 mg/kg as low dose quercetin group, and set middle (100 mg/kg) and high (200 mg/kg) dose group.

### 2.3. Animals and Treatment

**Figure 1:**
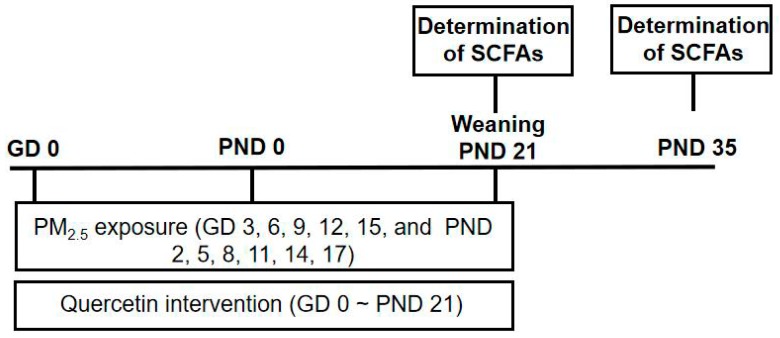
The experimental scheme of mouse maternal exposure to PM_2.5_ and received intragastric administration of quercetin. Maternal mice and offspring underwent further analysis after weaning.

8-Week-old Institute of Cancer Research (ICR) mice were obtained from the Department of Laboratory Animal Science of Peking University (Beijing, China) and housed under a temperature- and humidity-controlled specific pathogen-free animal facility with a 12-h/12-h light/dark cycle. Female mice were mated with healthy male mice overnight and were checked for vaginal plugs the next morning. The presence of a vaginal plug signified gestational day (GD) 0. Sixty pregnant ICR mice were divided into five groups, including normal control (NC) group, the PM_2.5_ group (PM_2.5_ group), and three quercetin intervention groups of low (50 mg/kg BW group), medium (100 mg/kg BW group), high (200 mg/kg BW group) dosage quercetin, 12 dams in each subgroup. Dams in the PM_2.5_ group and quercetin intervention groups were exposed to PM_2.5_ suspension (15.0 mg/kg) by intratracheal instillation on GD 3, 6, 9, 12 and 15 and postnatal day (PND) 2, 5, 8, 11, 14 and 17. Mice in the NC group were administered with the same amount of suspension from extracts of “blank” filter at the same time points. Meanwhile, the dams in the NC group and the PM_2.5_ group were given 0.15% CMCS and the dams in quercetin intervention groups were given different quercetin dosages of 50, 100, 200 mg/kg separately all by gavage lasting to PND 21. PM_2.5_ and quercetin intervention were only performed on dams, and sires were not received such intervention. All dams delivered naturally. On PND 3, litter size was adjusted to six with three male offspring and three female offspring. The method of selecting three females and three males per litter was as follows: all offspring of every litter were numbered and weighed, then three male and female mice were randomly selected using a random number table according to the weight of the pups. The breast feeding lasted to PND 21 and a pair of male and female offspring in the same litter were sacrificed on PND 21 and PND 35 separately. Dams were sacrificed on PND 21. The use of animals in this research was conducted in compliance with the Guidelines for Animal Research of Peking University (number of animal experimental ethical investigational tab: LA2016284). The treatment of the dams are listed in Table 1. After weaning, SCFAs of dams and offspring underwent further analysis (Figure 1). 

### 2.4. Determination of SCFAs

Each colon contents sample of dams on PND 21, offspring on PND 21 and 35 were collected immediately and stored at −80 °C until analyzed. SCFAs in colon (acetic acid, propanoic acid, butyric acid, isobutyric acid, valeric acid, and isovaleric acid) were quantified using gas chromatography (GC). (1) Reagent preparation: metaphosphoric acid (2.5000 ± 0.0050 g) was added to 100 mL sterilized water, and was used as the extraction liquid; croconic acid (0.6464 g) was added to 100 mL sterilized water, and was used as the internal standard substance; acetic acid (0.80 mLl), propanoic acid (0.60 mL), butyric acid (0.40 mL), isobutyric acid (0.40 mL), valeric acid (0.40 mL) and isovaleric acid (0.40 mL) were added to 100 mL sterilized water respectively, and were used as the individual standard substances; mixed the above standards into 100 mL sterilized water and was used as mixed standard substance. Metaphosphoric acid, croconic acid, acetic acid, propanoic acid, butyric acid, isobutyric acid, valeric acid and isovaleric acid were obtained from Sigma. (2) Pre-treatment process of samples: colon contents samples (150 mg) were added to 1.5 mL SCFAs extracting liquid and were shocked for 1 min, then samples were centrifuged at 12,000 rpm for 10 min to remove the solid material. The supernatant was frozen at −30 °C overnight. After 12 h, the supernatant was centrifuged again at 12,000 rpm for 10 min. After that supernatants (500 μL) were retained, then 100 μL crotonic acid solution was added as internal standard. The solution was mixed for 0.5 min, then filtered by through a 0.22 μm microporous membrane. (3) Samples were analyzed by GC: sample (1 μL) was injected into a GC (GC 2010 plus, Shimadzu, Kyoto, Japan), which was equipped with an Agilent DB-FFAP column (30 m × 0.32 mm × 0.50-μm film), Agilent 123-3233 (Agilent Technologies, Santa Clara, CA, USA), and a flame ionization detector. Nitrogen was the carrier gas. The GC temperature program was as follows: begin at 70 °C, increase to 180 °C at 15 °C/min, hold at 180 °C for 3 min, and then increase to 240 °C at 40 °C/min, hold at 240 °C for 5 min. Concentration of SCFAs were calculated using the internal standard method and expressed in mmol/L.

### 2.5. Statistical Analysis

Values were presented as the mean ± SD. The results were statistically analyzed using SPSS 17.0 software (SPSS, Inc., Chicago, IL, USA). Analysis of variance (ANOVA) was used to analyzed effects on SCFAs (acetic acid, propanoic acid, butyric acid, isobutyric acid, valeric acid and isovaleric acid). The least significant difference (LSD) post-hoc test was used if equal variance existed, or Tamhane’s T2 post-hoc test was used if equal variance did not exist. *P* < 0.05 was regarded as statistically significant, and all tests are two-sided.

## 3. Results

### 3.1. Acetic Acid

**Figure 2:**
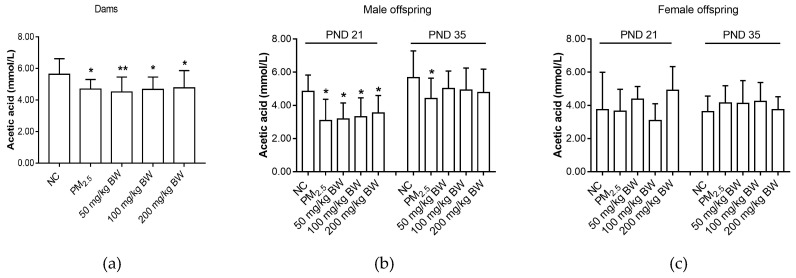
Acetic acid concentrations of dams and offspring. (**a**) Acetic acid of dams on PND 21. (**b**) Acetic acid of male offspring on PND 21 and PND 35. (**c**) Acetic acid of female offspring on PND 21 and PND 35. The data was expressed as mean ± SD of each group. Compared with the NC group, * indicates *p* < 0.05, ** indicates *p* < 0.01.

When compared with the NC group, the levels of acetic acid of dams and male offspring on PND 21 and PND 35 were lower in the PM_2.5_ group (*p* < 0.05) (Figure 2). 

### 3.2. Propanoic Acid

**Figure 3:**
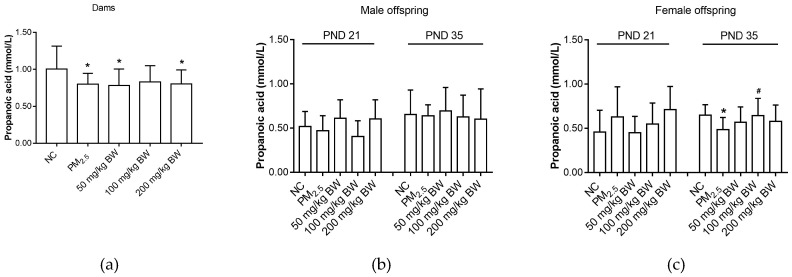
Propanoic acid concentrations of dams and offspring. (**a**) Propanoic acid of dams on PND 21. (**b**) Propanoic acid of male offspring on PND 21 and PND 35. (**c**) Propanoic acid of female offspring on PND 21 and PND 35. The data was expressed as mean ± SD of each group. Compared with the NC group, * indicates *p* < 0.05. Compared with the PM_2.5_ group, ^#^ indicates *p* < 0.05.

Maternal propanoic acid level in the PM_2.5_ group was lower than that in the NC group (*p* < 0.05). For male offspring, there was no difference among five groups (*p* > 0.05). Propanoic acid levels in female offspring on PND 35 in the PM_2.5_ group were lower than that in the NC group (*p* < 0.05). However, propanoic acid levels in the 100 mg/kg BW quercetin group were significantly higher compared with those in the PM_2.5_ group on PND 35 (*p* < 0.05) (Figure 3).

### 3.3. Butyric Acid 

**Figure 4:**
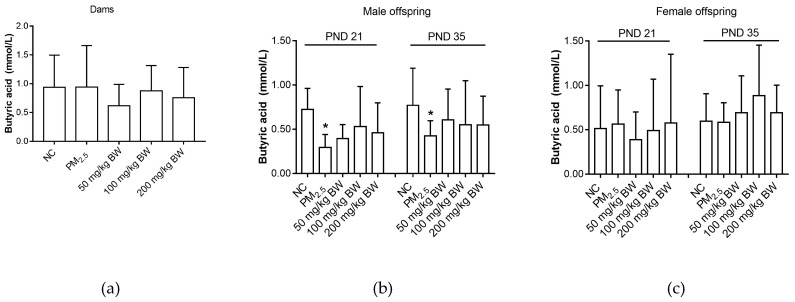
Butyric acid concentrations of dams and offspring. (**a**) Butyric acid of dams on PND 21. (**b**) Butyric acid of male offspring on PND 21 and PND 35. (**c**) Butyric acid of female offspring on PND 21 and PND 35. The data was expressed as mean ± SD of each group. Compared with the NC group, * indicates *p* < 0.05.

There was no difference among five groups in butyric acid levels (*p* > 0.05). Butyric acid of male offspring on PND 21 and PND 35 in the PM_2.5_ group were lower than those in the NC group (*p* < 0.05). However, for female offspring, there was no difference among five groups (*p* > 0.05) (Figure 4).

### 3.4. Isobutyric Acid

**Figure 5:**
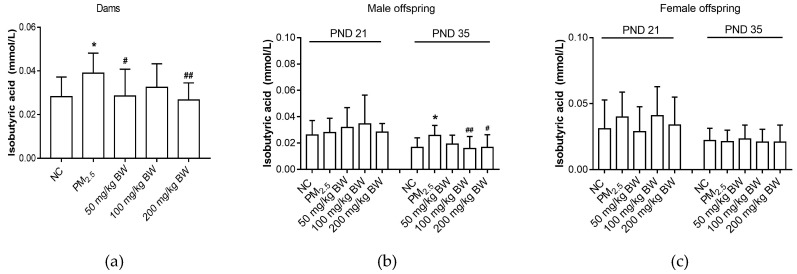
Isobutyric acid concentrations of dams and offspring. (**a**) Isobutyric acid of dams on PND 21. (**b**) Isobutyric acid of male offspring on PND 21 and PND 35. (**c**) Isobutyric acid of female offspring on PND 21 and PND 35. The data was expressed as mean ± SD of each group. Compared with the NC group, * indicates *p* < 0.05. Compared with the PM_2.5_ group, ^#^ indicates *p* < 0.05, ^##^ indicates *p* < 0.01.

Isobutyric acid levels in dams exposed to PM_2.5_ were higher than those in NC group dams (*p* < 0.05). Isobutyric acid levels of dams in the 50 and 200 mg/kg BW quercetin groups were significantly lower than that in the PM_2.5_ group (*p* < 0.05). Isobutyric acid levels in male offspring on PND 35 in the PM_2.5_ group were higher when compared with the NC group (*p* < 0.05). The isobutyric acid levels of male offspring on PND 35 in the 100 and 200 mg/kg BW quercetin intervention groups were significantly lower than that in the PM_2.5_ group (*p* < 0.05). However, for female offspring, there was no difference among five groups (*p* > 0.05) (Figure 5).

### 3.5. Valeric Acid 

**Figure 6:**
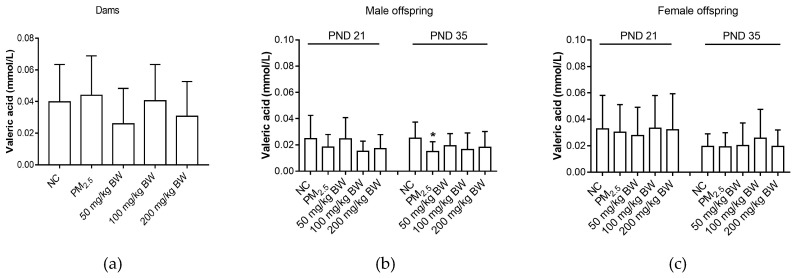
Valeric acid concentrations of dams and offspring. (**a**) Valeric acid of dams on PND 21. (**b**) Valeric acid of male offspring on PND 21 and PND 35. (**c**) Valeric acid of female offspring on PND 21 and PND 35. The data was expressed as mean ± SD of each group. Compared with the NC group, * indicates *p* < 0.05.

On PND 35, valeric acid concentrations of male offspring in the PM_2.5_ group were lower than those in NC group (*p* < 0.05). However, for dams and female offspring, there was no difference among five groups (*p* > 0.05) (Figure 6).

### 3.6. Isovaleric Acid

**Figure 7:**
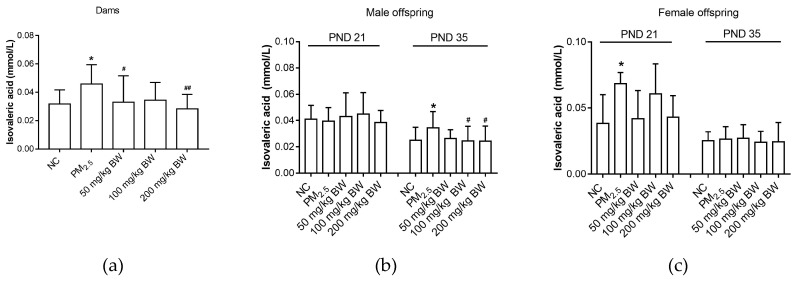
Isovaleric acid concentrations of dams and offspring. (**a**) Isovaleric acid of dams on PND 21. (**b**) Isovaleric acid of male offspring on PND 21 and PND 35. (**c**) Isovaleric acid of female offspring on PND 21 and PND 35. The data was expressed as mean ± SD of each group. Compared with the NC group, * indicates *p* < 0.05. Compared with the PM_2.5_ group, ^#^ indicates *p* < 0.05, ^##^ indicates *p* < 0.01.

As compared to the NC group, isovaleric acid of dams was higher in the PM_2.5_ group (*p* < 0.05). Isobutyric acid of dams in the 50 and 200 mg/kg BW quercetin group was significantly lower than that in the PM_2.5_ group (*p* < 0.05). Isovaleric acid levels of male offspring on PND 35 in the PM_2.5_ group were higher when compared with those in the NC group (*p* < 0.05). On PND 35, the isovaleric acid levels of male offspring in the 100 and 200 mg/kg BW quercetin groups were significantly lower than those in the PM_2.5_ group (*p* < 0.05). For female offspring, isovaleric acid levels on PND 21 in the PM_2.5_ group were higher when compared with NC group (*p* < 0.05) (Figure 7).

## 4. Discussion

Some studies have suggested that inhaled PM_2.5_ may influence gut microbiota [6]. However, the effects of maternal exposure to PM_2.5_ during pregnancy and lactation on SCFAs in the offspring are unknown. Additionally, there is no reported nutritional intervention to ameliorate the effects of PM_2.5_ exposure on SCFAs. In our study, animal model was used to investigate influences of PM_2.5_ exposure on SCFAs of the offspring. The results indicated that (1) PM_2.5_ exposure during pregnancy and lactation influenced SCFAs of the offspring; and that (2) quercetin administration might have the potential to offset the effects of mater PM_2.5_ exposure on SCFAs in the offspring.

Overall, concentrations of acetate acid, butyric acid, and propionate acid in the PM_2.5_ group were lower in dams and offspring mice when compared to those in the NC group in our study. Acetic acid, the dominant metabolites of bacteria fermentation, is involved in spleen, heart and brain metabolism. Thus, abnormally low level of acetic acid may affect the functions of these organs. Butyrate is the major and preferred metabolic fuel for colonocytes and provides at least 60–70% of the energy requirements. Accordingly, butyrate is necessary for the proliferation and differentiation of colonocytes [26,27], and reduction of butyric acid is commonly associated with a decrease in barrier function and increased susceptibility to mucosal inflammation. Propionic acid plays an important role in the regulation of blood glucose [28], as the decrease in propionic acid will affect the process of gluconeogenesis in the liver. 

We observed that concentrations of isobutyric acid and isovaleric acid in the PM_2.5_ group were higher than those in the NC group. Isobutyric acid and isovaleric acid originate from the degradation of amino acids valine, leucine or isoleucine. According, our findings in isobutyric acid and isovaleric acid may indicate a shift from a carbohydrate to a protein fermentation environment, which suggests changes in microbial composition. 

Our study revealed that SCFAs of offspring were influenced by maternal PM_2.5_ exposure. We speculated it may be related to the effects of PM_2.5_ on gut microbiota. It has been demonstrated that PM_2.5_ exposure could significantly increase gut microbial diversity [6]. Wang et al. found that chronic exposure to concentrated ambient PM_2.5_ significantly reduced the faecal bacterial ACE and Chao-1 estimators, and altered the composition of gut bacterial and fungal communities [29]. Because PM_2.5_ could influence the intestinal flora of dams and offspring mice, it is entirely posssible that PM_2.5_ exposure could also lead to the changes in SCFAs.

Due to economic and social impact factors, it is infeasible to solve PM_2.5_ pollution thoroughly in a short period. In this case, dietary intervention may be useful to offset the detrimental effects of PM_2.5_ exposure. Naturally bioactive compounds are widely recognized to have potential biological properties [30,31]. Overall, it is estimated that around 5%–10% of the total dietary polyphenols are absorbed in the small intestine, while the rest reaches the colonic region unabsorbed. Unabsorbed bioactive compounds could be metabolized by gut microbiota and give rise to metabolites with a variety of physiological roles [32]. Quercetin, a kind of polyphenol, had been reported to show no maternal or fetal toxicity even with a safe daily intake of 2000 mg/kg body weight during gestation in rats [33]. Etxeberria et al. found that supplementation with quercetin, although not significant, was found to slightly increase concentrations of acetate, propionate and butyrate when compared to the high-fat sucrose diet fed rats [34]. Considering the multiple effects of quercetin, we attempted to investigate quercetin as an intervention substance. Based on previous studies [14,22,35], we tested the following three doses of quercetin: 50, 100 and 200 mg/kg. We found that quercetin could adjust the propanoic acid of the female through increasing concentration of it. In quercetin groups, levels of isobutyric acid and isovaleric acid were lower than that in the PM_2.5_ group. It was worth noting that quercetin intervention was ineffective to restore the level of acetic acid, butyric acid and valeric acid. These above results indicated that quercetin had limited improvement effects. 

Our study showed sex-dependent effects of PM_2.5_ maternal exposure and quercetin intervention on offspring’s SCFAs. These differences were mainly reflected in the following aspects: (1) the influences of PM_2.5_ on offspring of two sexes were different, showing greater effects on male offspring (for instance, acetic acid, butyric acid, isobutyric acid, valeric acid of male offspring were more influenced than that of female offspring); (2) the period of influence was different for the offspring of different sexes (the effect on isovaleric acid was mainly reflected on PND 35 for male offspring and on PND 21 for female offspring; (3) for the intervention of quercetin, different sexes of offspring mice showed different effects (quercetin decreased isovaleric acid levels in male offspring but not in female offspring). 

Sex differences in the SCFAs may suggest that offspring sex is an important host factor in the metabolic process. Sex differences of SCFAs maybe related with immunological tolerance or metabolic status, which needs further study. Our study in 2018 found that mice exposed to maternal PM_2.5_ exposure could influence the weight and coefficient of major organs and small intestine length of the offspring in a sex-specific manner [36]. For male offspring, weight of spleen and kidney were lower in PM_2.5_ group than that in control group, but there was no significant difference among female mice in each group. Small intestine and large intestine of male offspring were shorter in PM_2.5_ group compared with control group, and there was no significant difference among female mice in each group. Another previous study in 2018 also showed sex differences in the behavioral effects of PM_2.5_ exposure on offspring [22]. Morris water maze test showed cognitive impairment in female offspring mice, and there was no significant difference among male mice in each group. Meanwhile, the results of TNF-α in serum and hippocampus in both gender shows inconsistency. Therefore, sex should indeed be taken into account in the impact of maternal PM_2.5_ exposure on offspring health. 

Although the present study demonstrated a marked impact of maternal PM_2.5_ exposure on the SCFAs of offspring and suggested that quercetin could have certain ameliorative effects, several limitations should be noted. Firstly, while ingestion of ambient particles was shown to alter concentrations of SCFAs, the mechanism of how PM_2.5_ inhalation impact them remains to be determined. Secondly, given the different physiology between humans and mice, human studies are needed to confirm the specialized role of maternal PM_2.5_ exposure in the metabolism process of SCFAs of offspring. Thirdly, the reasons for the differences between male and female mice are unclear and need further investigation. Our future studies will focus on the adverse outcomes of offspring associated with SCFAs changes. We will explore the incidence of obesity and diabetes in the offspring when the mother is exposed to PM_2.5_, meanwhile, the mitigating effect of quercetin on these adverse effects will be investigated. Acetic acid, butyric acid and propionic acid are related to insulin sensitivity and energy metabolism, thus effects on SCFAs caused by PM_2.5_ will influence the occurrence and development of diseases related to glucose metabolism and energy metabolism. Additionally, we will explore the effects of PM_2.5_ maternal exposure and quercetin intervention on the occurrence of intestinal diseases such as enteritis in the offspring. Butyrate is necessary for the proliferation and differentiation of colonocytes. The reduction of butyrate caused by maternal PM_2.5_ exposure is likely to affect intestinal function and cause enteritis. The effects on SCFAs could suggest some undesirable outcomes in offspring that may be caused by maternal PM_2.5_ exposure.

## 5. Conclusions

In conclusion, PM_2.5_ tracheal exposure during gestation and lactation could influence SCFAs of offspring. Quercetin administration might have the potential to offset the adverse effects of mater PM_2.5_ exposure on SCFAs in the offspring to some extent. The above effects were showed in a sex-dependent manner.

## Figures and Tables

**Table 1:** Animal treatment.

Group	N	PM_2.5_ (mg/kg)	Quercetin (mg/kg)
NC	12	-	-
PM2.5	12	15	-
50 mg/kg BW quercetin	12	15	Quercetin (50)
100 mg/kg BW quercetin	12	15	Quercetin (100)
200 mg/kg BW quercetin	12	15	Quercetin (200)
